# District-level ecological analysis of the association between stunting and infectious diseases among children under 2 years in Rwanda, 2020–2024

**DOI:** 10.3389/fpubh.2026.1855873

**Published:** 2026-08-04

**Authors:** Seleman Ntawuyirushintege, Nouh Saad Mohamed, Ayman Ahmed, Muhammed Semakula, Eric Remera, Fabrizio Tediosi, Kaspar Wyss

**Affiliations:** 1 College of Medicine and Health Sciences, University of Rwanda, Kigali, Rwanda; 2 Department of Implementation, Swiss Tropical and Public Health Institute (Swiss TPH), Allschwil, Switzerland; 3 Medical Faculty, University of Basel, Basel, Switzerland; 4 Pan-Africa One Health Institute (PAOHI), Kigali, Rwanda; 5 Institute of Endemic Diseases, University of Khartoum, Khartoum, Sudan; 6 Ministry of Health, Kigali, Rwanda; 7 Division of Research Innovation and Data Science, Rwanda Biomedical Centre, Kigali, Rwanda; 8 University of Milan, Milano, Italy

**Keywords:** children under 2 years, diarrhea, infectious diseases, malaria, respiratory infections, Rwanda, schistosomiasis, stunting

## Abstract

**Background:**

Stunting among children under 2 years remains a major public health problem in Rwanda despite substantial investments in nutrition, water, sanitation, and hygiene (WASH), and maternal-child health. Infectious diseases, particularly diarrheal diseases, malaria, respiratory infections, and schistosomiasis, significantly contribute to growth faltering; however, their association and interconnectivity with stunting in Rwanda have not been comprehensively examined.

**Methods:**

This district-level ecological study analyzed data from 2020 and 2022-–2024 by combining aggregated stunting prevalence from the national nutrition surveillance system with facility-reported infectious disease data from the District Health Information System-2. Stunting and infectious disease records were analyzed. Descriptive statistics, spatial analyses, Pearson’s correlation, and multivariable linear regression were performed to assess district-level associations between stunting and selected infectious diseases, while adjusting for population density and maternal education.

**Results:**

National stunting prevalence declined from 33.1% in 2020 to 21.7% in 2024. At the district level, Rubavu had the highest stunting prevalence (35.0%), whereas Nyarugenge recorded the lowest (6.0%). No significant correlations were observed between stunting and infectious diseases during 2020–2023; however, in 2024, diarrheal diseases showed a significant positive correlation with stunting (r = 0.477, *p* < 0.05). The correlations varied widely by district. Malaria showed a significant positive correlation with stunting in Rutsiro, Musanze, Nyamagabe, Nyaruguru, Rubavu, and Kamonyi, with Pearson’s r values of 0.96, 0.93, 0.91, 0.90, 0.85, and 0.82, respectively (*p* < 0.05). Diarrheal diseases were associated with stunting in Gisagara District, with a Pearson’s r of 0.98 (*p* < 0.05). Respiratory diseases were positively associated with stunting in Nyamasheke (Pearson’s r = 0.94, *p* < 0.05) but negatively associated in Nyarugenge, Kamonyi, and Muhanga (Pearson’s r = −0.98, −0.96, and −0.96, respectively). In multivariable analysis, none of the infectious disease indicators were significantly associated with district-level stunting prevalence, while population density showed a strong inverse association (*β* = −0.0052, *p* < 0.001).

**Conclusion:**

District-level patterns of stunting in Rwanda were not consistently associated with facility-reported infectious diseases. Since the data on stunting and infections were aggregated and not linked to the same individual child, these findings should be interpreted as ecological associations rather than evidence of causality. The results support the need for ongoing multisectoral interventions in nutrition, WASH, maternal and child health, and district-tailored infection control, particularly in persistently high-burden areas.

## 1 Introduction

Childhood stunting, defined as a height-for-age more than two standard deviations below the World Health Organization (WHO) growth standards, remains a significant public health concern in low- and middle-income countries (1). Globally, it affects an estimated 150 million children under 5 years of age, with the highest burden in sub-Saharan Africa, including Rwanda (2). Stunting not only reflects chronic undernutrition but also translates into long-term consequences for physical growth, cognitive development, educational attainment, and economic productivity in adulthood (3). The “First 1,000 Days” (from conception to a child’s second birthday) is now recognized as a critical window of opportunity. Nutritional deficiencies during this period can lead to irreversible growth and developmental impairments. Indeed, approximately half of the instances of linear growth faltering is estimated to originate between conception and the first 6 months of life (4, 5).

In Rwanda, substantial progress has been made in improving child health and nutrition over the past 5 years, with the percentage of children under 2 years of age affected by stunting decreasing from 33.1% in 2020 to 21.7% in 2024; however, stunting among children under 2 years of age remains a significant issue (4). National surveys conducted between 2020 and 2024 indicate that approximately one in three children in this age group experience stunted growth, reflecting persistent challenges related to food security, maternal nutrition, and childcare practices (4).

Infectious diseases are a well-documented contributor to stunting (6). Recurrent or severe infections can impair nutrient absorption, increase metabolic demands, and reduce appetite, collectively undermining optimal growth (7). For instance, increased incidence of malaria infection among < 2 years in Ethiopia showed increased risk of becoming stunted or wasted (8). Moreover, in Mali, severe stunting was associated with malaria incidence (9). Further studies on the effect of malaria on stunting generally found higher odds of stunting among children with malaria, including asymptomatic infections (10–12). Meanwhile, for respiratory diseases, stunting was found to worsen the outcomes of respiratory infections. In several studies, stunted children had higher treatment failure rates and longer recovery compared to non-stunted peers (13–15).

In relation to diarrheal diseases, children under 2 years of age who experience repeated episodes of moderate-to-severe diarrhea, particularly due to Shigella infections, showed significant drops in their height-for-age z-scores (HAZ) and increased odds of becoming stunted (16–19). Furthermore, schistosomiasis, particularly infections caused by *Schistosoma mansoni*, was linked to inadequate dietary fat intake among children and was found to play a significant and independent role in increasing the risk of developing stunting when compared to uninfected children (20–22).

In Rwanda, diseases such as malaria, diarrheal infections, acute respiratory infections, and schistosomiasis are common among young children and have been linked to growth retardation (23). Malaria, for instance, contributes to anemia and systemic inflammation, while repeated diarrheal episodes interfere with nutrient uptake and gut health. Schistosomiasis, although less commonly studied in children under 2 years, may exacerbate malnutrition through chronic blood loss and immune modulation (23).

Understanding the relationship between stunting and infectious disease patterns in early childhood is crucial for informing public health interventions. Despite improvements in healthcare access and vaccination coverage, the persistent prevalence of stunting in Rwanda raises the question of the interlinkages between infectious disease patterns and nutritional status. Investigating the interplay between infectious diseases and stunting among Rwandan children under 2 years of age will provide evidence to guide multisectoral approaches aimed at reducing the burden of growth faltering and its long-term consequences in Rwanda and other LMICs.

## 2 Methods

### 2.1 Study design

This retrospective correlational study aimed to assess the association between stunting and selected infectious diseases, specifically malaria, schistosomiasis, diarrheal diseases, and acute respiratory infections, among children aged below 2 years in Rwanda. The research analyzed routinely collected district-level data from 2020 to 2024. The study examined whether variations in stunting prevalence among children under 2 years correlate with the different infectious diseases reported at the health center level over the same period.

### 2.2 Study population

**Figure 1:**
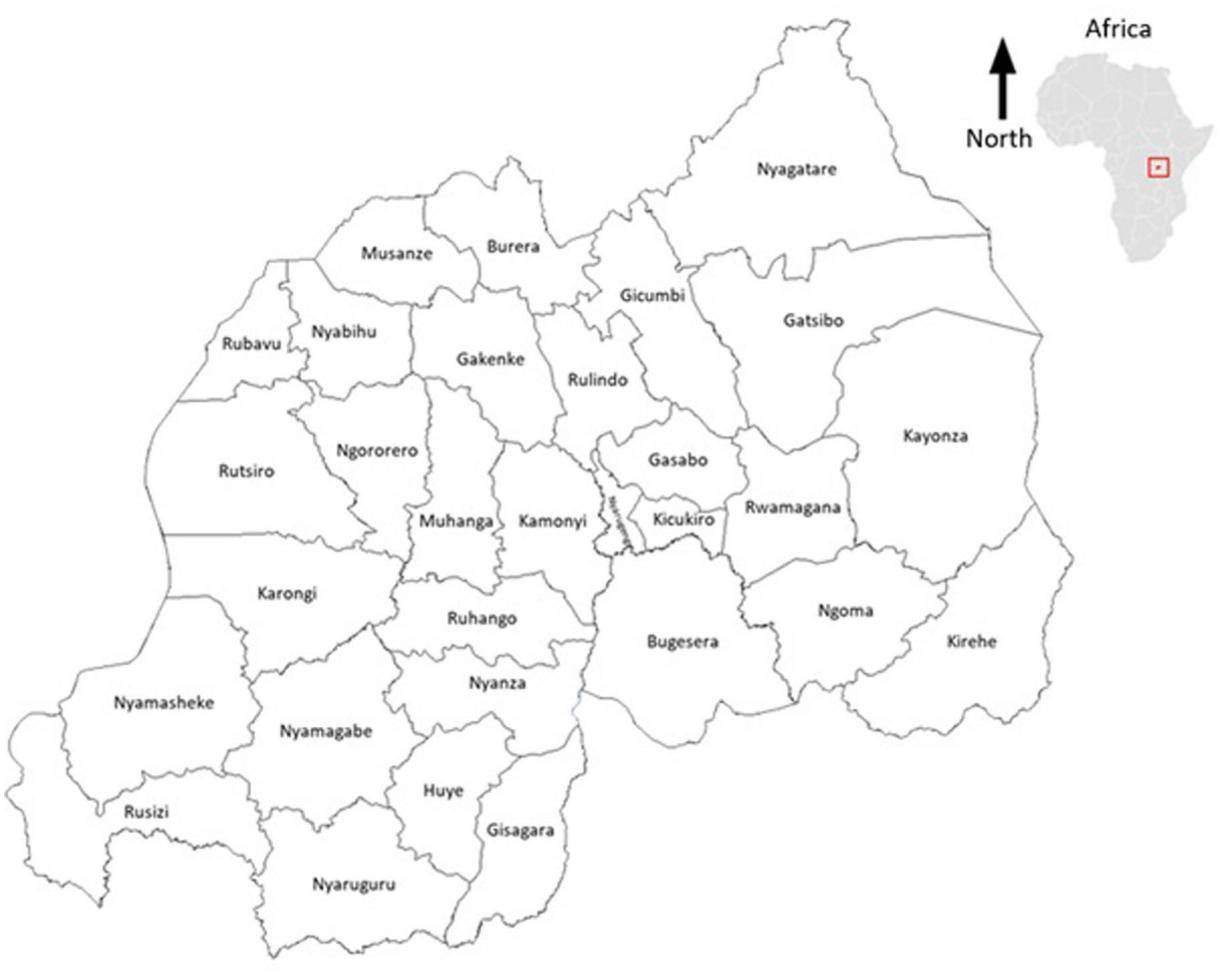
Geographic distribution and boundaries of Rwanda’s administrative districts.

The study population comprised all children aged 0–23 months who attended health facilities in Rwanda during 2020 and 2024. The focus has been on the under-two population per administrative district, linked with reported cases of malaria, schistosomiasis, diarrheal diseases, and respiratory infections among the same age group. Rwanda is administratively divided into 4 provinces, the city of Kigali, 30 districts, 416 sectors, 2,148 cells, and 14,837 villages. Each district is responsible for aggregating and reporting health data from public and private health facilities through the national Health Management Information System (HMIS). The geographical distribution and boundaries of these administrative districts are illustrated in Figure 1.

### 2.3 Data sources and variables

Data on stunting prevalence among children aged 0–23 months were obtained from the national nutrition surveillance system under the Maternal, Child, and Community Health (MCCH) program of the Rwanda Ministry of Health.^1^ This dataset includes anthropometric indicators routinely reported by health centers and aggregated at sector, district, and national levels. For children aged 0–23 months, nutritional surveillance used recumbent length measurement, consistent with the WHO Child Growth Standards, and stunting was defined as a length-for-age z-score (LAZ) < −2 standard deviations from the median (24). The data series used in this study did not include the year 2021 because the stunting surveillance system was suspended during the COVID-19 pandemic.

The data regarding the number of cases of infectious diseases among children < 2 years, including malaria, schistosomiasis, diarrheal diseases, and acute respiratory infections, were sourced from the District Health Information System 2 (DHIS-2). The DHIS-2 compiles routine monthly reports from all public and most private health facilities.

The two datasets and district figures were combined and aggregated for further analysis. Additional covariates captured district-level characteristics, such as sanitation coverage (households with improved sanitation), population density (persons per km^2^), maternal education (percentage of mothers with at least primary education), and health service coverage (proportion of children accessing key child health services such as vaccination and antenatal care visits).

Data cleaning involved consistency checks by matching district numbers reported in the DHIS-2 to those in the Ministry of Health reports, and missing values were addressed by collating them from the Standard of Care’s paper-based reports. The reported figures were validated against the Ministry of Health’s annual summary reports. The final dataset was reviewed and approved in consultation with the MCCH and HMIS teams at the Ministry of Health.

### 2.4 Statistical analysis

Data analysis was conducted using R software (version 4.5.1) (25). Descriptive statistics were performed to summarize the distribution of key indicators across years and geographical units. Measures including annual mean cases, standard deviation, and interquartile range were calculated to describe the prevalence of stunting and the reported cases of malaria, schistosomiasis, diarrheal diseases, and respiratory infections among children under 2 years. For infectious disease indicators, facility-based consultation proportions were calculated by dividing the number of reported cases of malaria, schistosomiasis, diarrheal disease, or respiratory infections among children under 2 years by the total number of children under 2 years who consulted health facilities in the same district and year. These indicators, therefore, reflect the disease burden among children who accessed health services and should not be interpreted as population prevalence. Time-series plots were used to illustrate changes in stunting and disease prevalence from 2020, and between 2022 and 2024, while choropleth maps and heatmaps were generated in R using ggplot2 (26), tmap (27), corrplot (28), and sf packages (29, 30) to display spatial heterogeneity and potential clustering at the district level.

To assess the relationship between stunting and infectious diseases, Pearson’s correlation coefficient analysis was conducted. The association of infectious diseases with stunting for each district was calculated across multiple years (2020, 2022–2024) to examine how temporal changes in disease prevalence, including malaria, diarrhea, respiratory infections, and schistosomiasis, among children under 2 years are associated with stunting. National-level annual means were used to explore overall temporal associations across the study period. Pearson’s correlation coefficients with *p*-values < 0.05 were considered statistically significant.

To further explore the associations and account for potential confounding factors, multivariable regression analyses were performed. Specifically, linear regression models were used to estimate the effect of each infectious disease indicator on stunting prevalence at the district level. In our analysis, we adjusted for covariates such as population density, sanitation coverage, and maternal health education by including aggregated data on the number of healthcare visits, frequency and duration of breastfeeding, vaccination chart, and utilization of impregnated bed nets at the district level. These data were obtained from the national nutrition surveillance system, which has reported a protective role in reducing child exposure to infectious diseases (31). In addition to the collinearity assessment, predictors were examined for near-zero variance before inclusion in the final multivariable model. Variables with very limited variation across districts and years were considered unsuitable for primary regression analysis because they provide limited statistical information and may produce unstable estimates. Since schistosomiasis was biologically plausible, it was retained for descriptive analysis but excluded from the primary multivariable regression model.

Before fitting the final model, multicollinearity among candidate predictors was assessed using a predictor correlation matrix, variance inflation factors (VIFs), and tolerance values. Variables with evidence of high collinearity, defined as VIF > 5 or tolerance < 0.20, were considered for exclusion from the final model. Sanitation coverage and health service coverage were excluded from the final model because of high collinearity with other district-level development and service-access indicators. Model assumptions were assessed using residual-versus-fitted plots, Q–Q plots of standardized residuals, and checks for influential observations. Regression coefficients were reported with standard errors, 95% confidence intervals, and *p*-values. Both R^2^ and adjusted R^2^ were reported to describe model fit, with adjusted R^2^ used to account for the number of predictors relative to the available district-level observations.

All statistical tests were two-tailed, and statistical significance was set at a *p*-value of < 0.05. Regression coefficient results were presented with 95% confidence intervals. Standardized coefficients were used to facilitate the comparison of effect sizes across different diseases, where appropriate. Sensitivity analyses were conducted by excluding sectors with incomplete reporting or extremely small populations, and by comparing results from different model specifications.

### 2.5 Ethics approval

This study is based on a secondary analysis of existing, de-identified data, with no primary data collection and no direct interaction with human participants. Therefore, no additional ethical approval was required for the present analysis in accordance with national regulations prevailing in Rwanda and institutional policies. The original data collection procedures were reviewed and approved by the relevant ethics committees and institutional review boards, and were conducted in compliance with the Declaration of Helsinki. For the present secondary analysis of anonymized data, no informed consent was required.

## 3 Results

### 3.1 Stunting and infectious diseases prevalence in Rwanda districts

**Figure 2:**
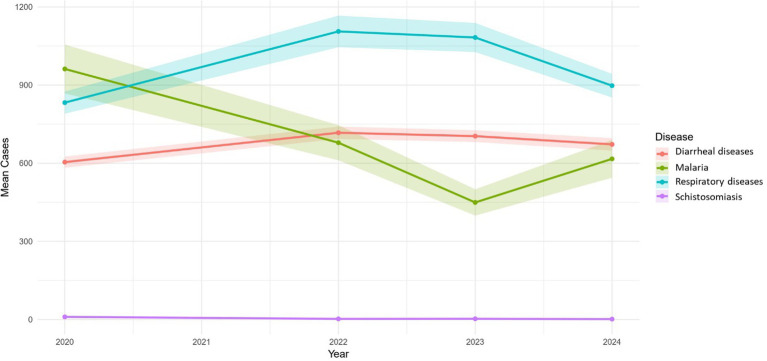
Annual mean facility-reported infectious disease cases among children under 2 years in Rwanda between 2020 and 2022–2024.

Between 2020 and 2024, the national prevalence of stunting among children aged 0–23 months decreased from 33.1% (95% CI: 33.0–33.3%) to 21.7% (95% CI: 20.9–22.1%), representing a 34.4% (95% CI: 33.6–36.7%) reduction (4) (Supplementary Tables S1, S2). For the diseases investigated, respiratory infections consistently had the highest mean number of cases across all years, peaking around 2022 before declining slightly in 2024. Malaria cases declined steadily from 2020 to 2023, with a modest rebound in 2024. Diarrheal disease cases remained relatively stable, with a gradual increase from 2020 to 2022 followed by a slight decline thereafter. In contrast, schistosomiasis reports the lowest mean case numbers, remaining nearly negligible throughout the study period (Figure 2; Supplementary Table S3).

**Figure 3:**
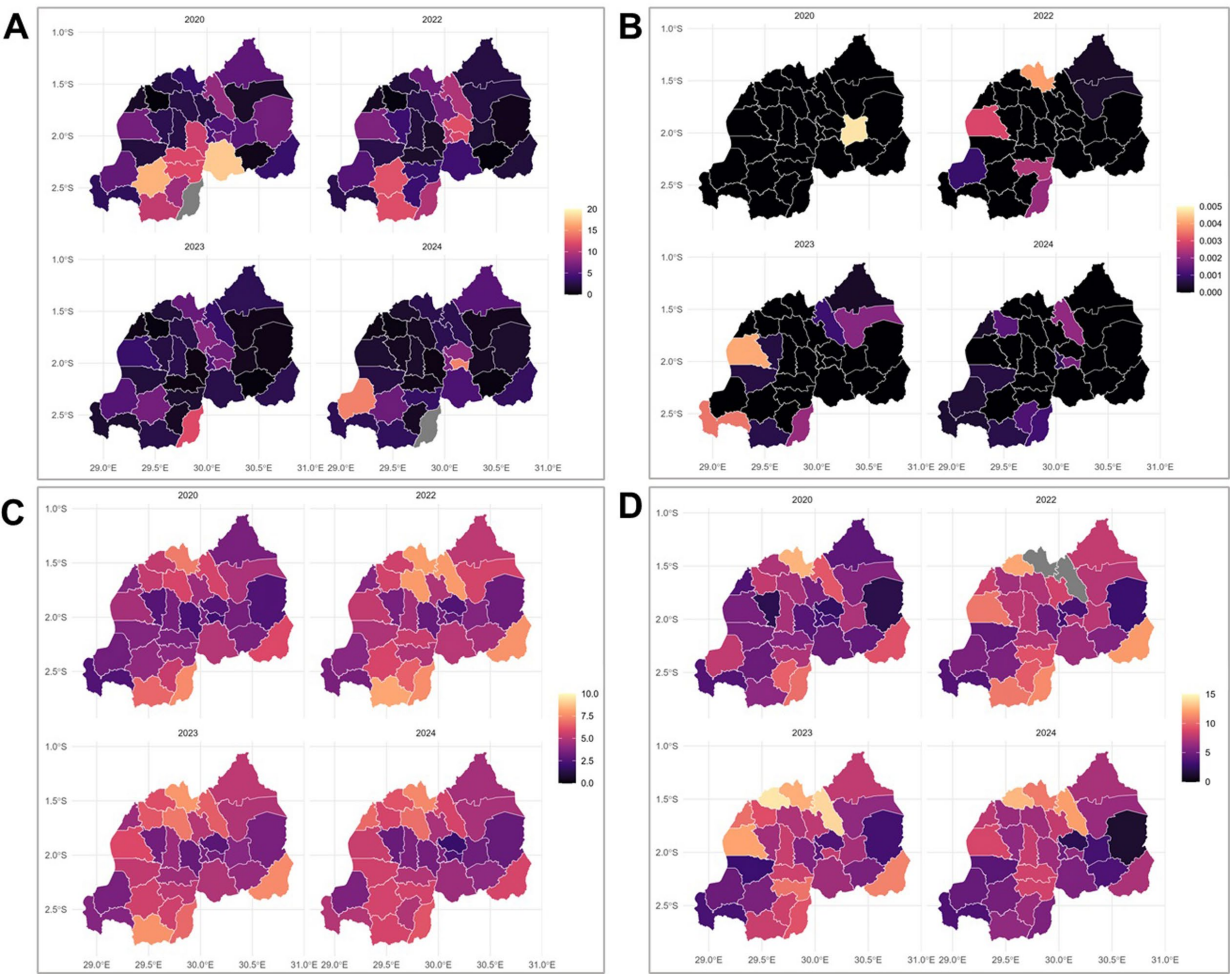
Facility-reported infectious disease consultation proportions among children under two years across Rwanda’s districts during 2020 and 2022–2024. **(A)** Malaria infections proportion, **(B)** Schistosomiasis proportion, **(C)** Diarrheal diseases proportion, and **(D)** Respiratory diseases proportion.

The highest burden of malaria consistently appeared in Gisagara, where the facility-reported malaria consultation proportion among children under 2 years consulting a health service peaked at 33.1% in 2020 and again reached 28.4% in 2024, indicating persistent malaria transmission in the southern region. Other districts such as Nyamagabe (16.8% in 2020) and Nyanza (12.0% in 2020) also showed relatively high rates. In contrast, the lowest prevalence was observed in districts such as Nyabihu (0.2%), Ngoma (0.7%), and Rubavu (1.3%). Over time, facility-reported malaria consultations among children accessing a health service generally declined across most districts. For example, in Bugesera, facility-reported malaria consultation among < 2 years reduced from 17.8% in 2020 to 4.8% in 2024, and in Kamonyi from 11.1 to 0.5% (Figure 3A). Meanwhile, schistosomiasis showed the lowest reported burden among the selected infectious diseases. The majority of districts reported zero cases across the study years, and the few reported cases were sparse and geographically limited (Figure 3B).

Facility-reported diarrheal disease prevalence among children < 2 years showed moderate variation across districts and years. The highest prevalence was reported in Burera, rising from 7.2% in 2020 to 8.0% in 2022, and remaining elevated to 7.5% in 2024. Other districts, such as Nyaruguru and Rutsiro, also demonstrated higher prevalence rates (up to 8.2% in 2022 and 6.9% in 2024, respectively). Central and eastern districts such as Gasabo, Kamonyi, and Rwamagana recorded lower diarrheal prevalence (typically 2–4%) (Figure 3C). However, facility-reported respiratory infection prevalence among children consulting health services was consistently higher than that of diarrheal diseases across most districts. The northern districts, particularly Burera (12.6% in 2020; 16.2% in 2022), Gicumbi (15.7% in 2022), and Musanze (12.3–14.3% between 2022 and 2023), recorded the highest rates. In contrast, Gasabo, Kayonza, and Ngoma maintain lower levels (typically below 4–5%) (Figure 3D).

### 3.2 Association between infectious diseases and stunting

**Figure 4:**
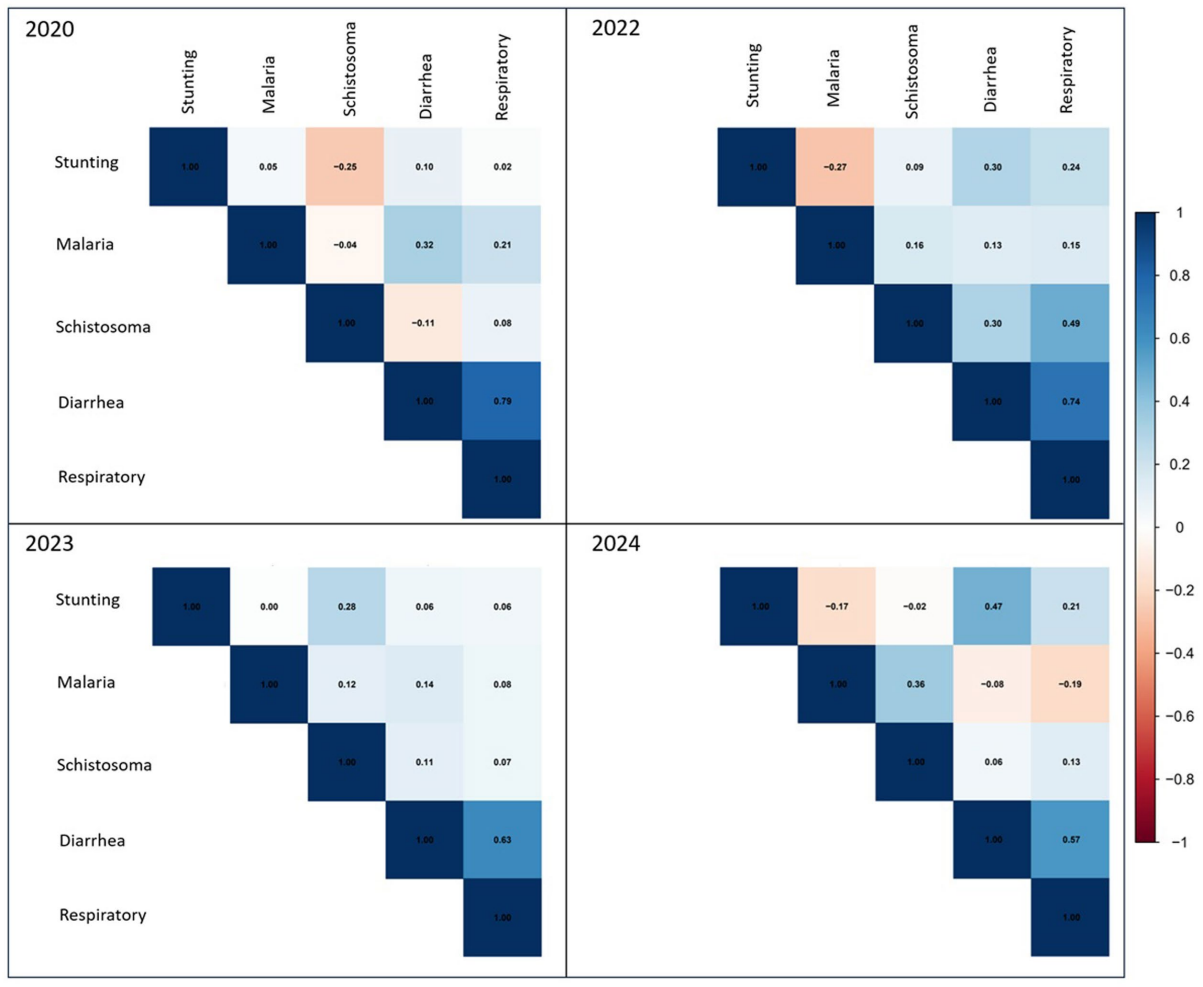
National-level ecological correlations between stunting prevalence and facility-reported infectious disease indicators among children under 2 years in Rwanda during 2020 and 2022–2024.

In 2020, 2022, and 2023, stunting showed no significant positive or negative correlation with diarrheal diseases, malaria, respiratory infections, or schistosomiasis, as all associations were statistically insignificant (*p* > 0.05). However, in 2024, stunting demonstrated a moderately positive and statistically significant correlation with diarrheal diseases (r = 0.477, *p* < 0.05) (Figure 4).

Across districts, malaria showed the strongest positive association with stunting in Rutsiro (r = 0.96), Musanze (r = 0.93), Nyamagabe (r = 0.91), Nyagatare (r = 0.90), Rubavu (r = 0.85), Bugesera (r = 0.84), and Kamonyi (r = 0.82), while the strongest negative correlations were observed in Kicukiro (r = −0.95) and Gasabo (r = −0.84). For schistosomiasis, the highest positive associations were found in Rwamagana (r = 0.57), Nyagatare (r = 0.30), and Nyanza (r = 0.24), whereas strong negative correlations were noted in Gicumbi (r = −0.91), Kicukiro (r = −0.80), Nyabihu (r = −0.85), and Nyagatare (r = −0.89). Although the reported schistosomiasis cases were extremely low across most districts and study years, their distribution was examined carefully before inclusion in inferential models since schistosomiasis was among the candidate infectious disease indicators because of its biological plausibility as a contributor to undernutrition and impaired child growth, as well as its relevance to national infectious disease surveillance.

**Figure 5:**
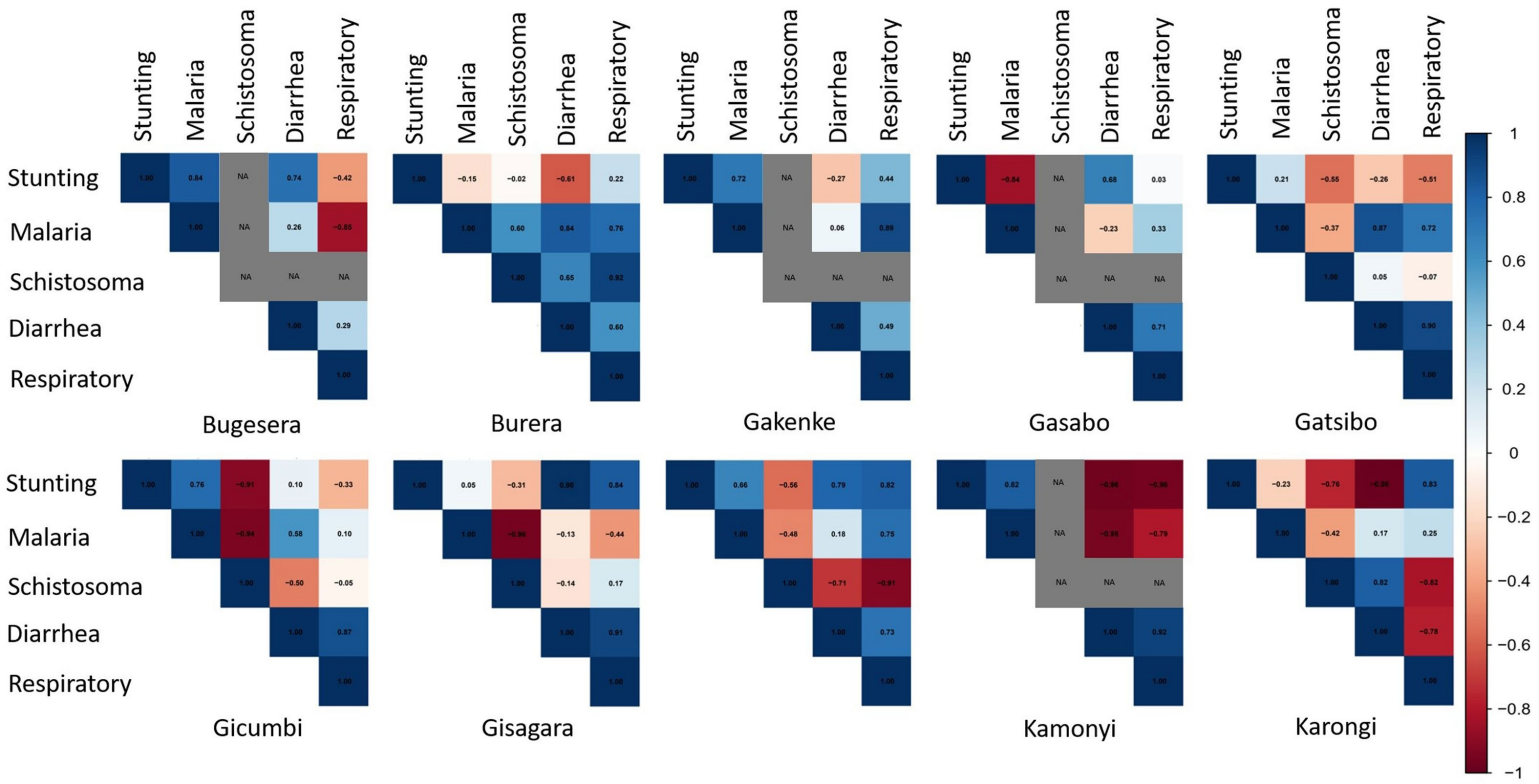
District-level ecological correlations between stunting prevalence and facility-reported infectious disease indicators among children under 2 years across Rwanda’s districts during 2020 and 2022–2024.

**Figure 6:**
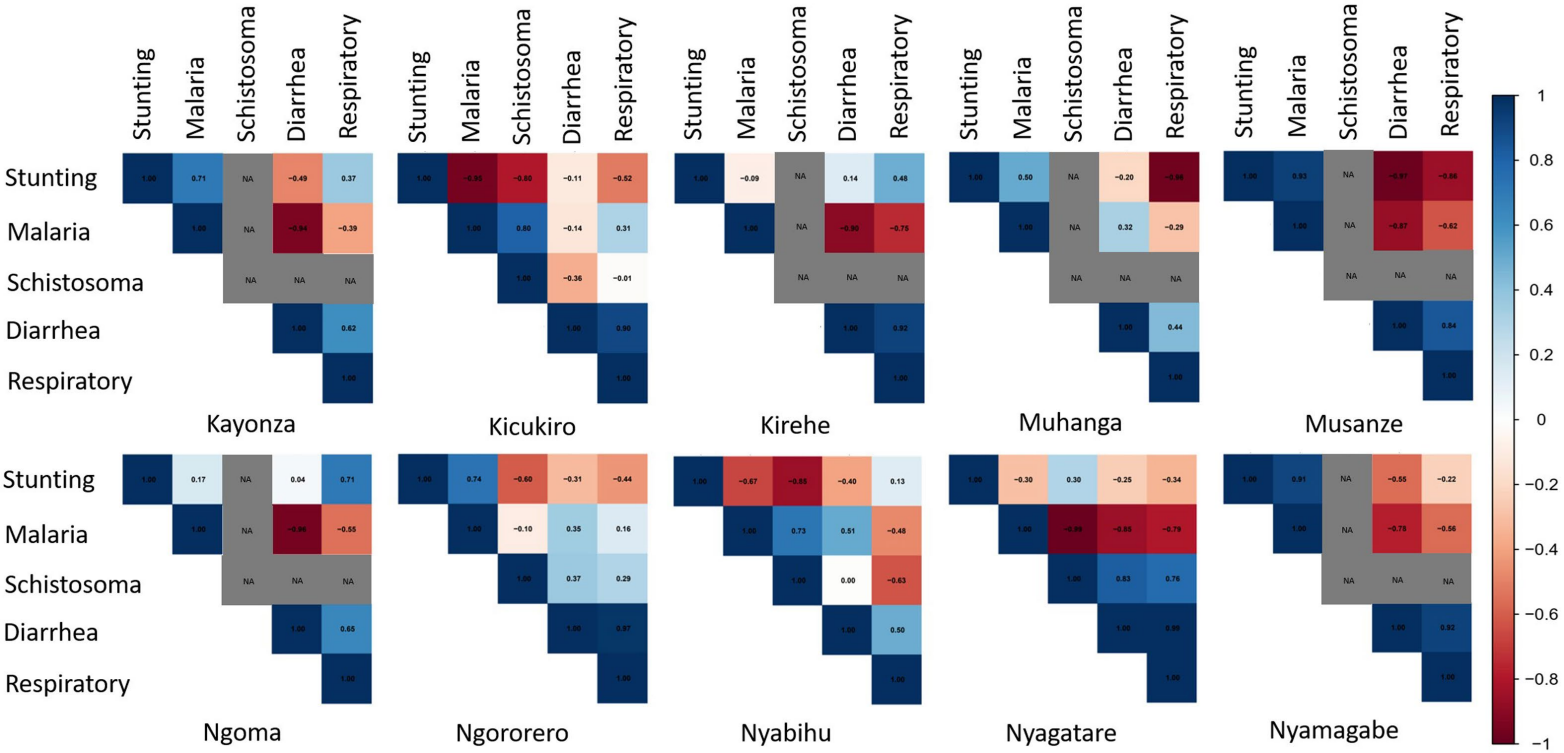
District-level ecological correlations between stunting prevalence and facility-reported infectious disease indicators among children under 2 years across Rwanda’s districts during 2020 and 2022–2024.

**Figure 7:**
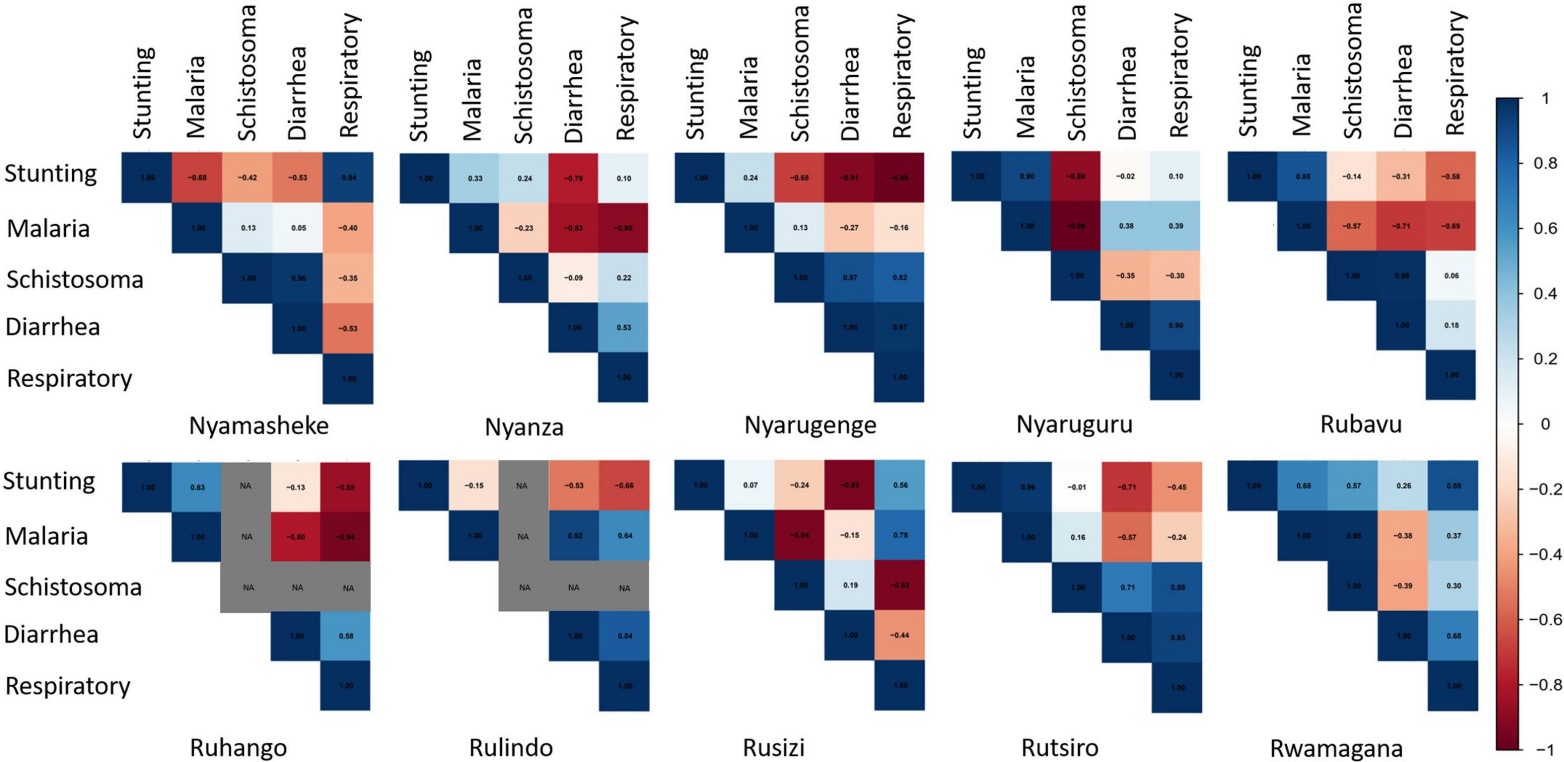
District-level ecological correlations between stunting prevalence and facility-reported infectious disease indicators among children under 2 years across Rwanda’s districts during 2020 and 2022–2024.

Diarrheal diseases exhibited the strongest positive correlation with stunting in Gisagara (r = 0.98), while pronounced negative associations were seen in Karongi (r = −0.98), Kamonyi (r = −0.98), Musanze (r = −0.97), Rusizi (r = −0.93), and Nyarugenge (r = −0.91). Similarly, respiratory infections were most positively associated with stunting in Nyamasheke (r = 0.94), whereas the strongest negative correlations were observed in Nyarugenge (r = −0.98), Kamonyi (r = −0.96), and Muhanga (r = −0.96) (Figures 5–7). These strong negative correlations should be interpreted with caution because they reflect district-level ecological patterns and may be influenced by shared contextual factors, such as urbanization, infrastructure, healthcare access, WASH conditions, and socioeconomic differences, rather than direct biological relationships between infections and stunting.

### 3.3 Multivariable analysis of the relationship between stunting and infectious diseases

After adjusting for population density, sanitation, and maternal education, the multivariable regression model explained 44.8% of the variability in district-level stunting prevalence (adjusted R^2^ = 0.33, *p* = 0.0069). The reduction from R^2^ to adjusted R^2^ indicates that some of the model’s explanatory value decreased after accounting for the number of predictors relative to the available district-level observations; therefore, model fit should be interpreted cautiously. Malaria, diarrheal diseases, and respiratory infections were included as infectious disease indicators in the primary model. Schistosomiasis was not retained because most districts reported zero cases across the study period, resulting in near-zero variance and limited inferential value. None of the retained infectious disease indicators was significantly associated with district-level stunting prevalence (*p* > 0.05). Population density was inversely and significantly associated with stunting (*β* = −0.0052, *p* < 0.001), suggesting that urbanized districts experience lower rates of child stunting. Sanitation coverage and health service coverage were excluded from the final model due to collinearity (Table 1).

**Table 1:** Multivariable analysis of the relationship between stunting among under 2 years children and infectious diseases in Rwanda in 2020, and 2022–2024.

Predictor	Estimate (β)	Std. error	95% CI	t value	*p*-value	VIF	Tolerance
(Intercept)	3.585	3.127	-2.8200 to 9.9900	1.146	0.261	-	-
Malaria	−0.000028	0.000018	−0.0001 to 0.0000	−1.59	0.124	1.087	0.920
Schistosomiasis	−0.0123	0.0084	−0.0294 to 0.0049	−1.47	0.153	1.059	0.945
Diarrheal	−0.000024	0.000036	−0.0001 to 0.0001	−0.68	0.504	2.547	0.393
Respiratory	0.000017	0.000013	0.0000 to 0.0000	1.33	0.195	2.361	0.424
Population density per km^2^	−0.00517	0.00128	−0.0078 to −0.0025	−4.03	0.0004	1.185	0.844
Maternal education	−0.0184	0.1101	−0.2438 to 0.2071	−0.17	0.869	1.132	0.883

## 4 Discussion

This district-level analysis highlights significant reductions in child stunting in Rwanda from 2020 to 2024, a trend that may be associated with the country’s comprehensive and coordinated national strategies (32, 33). The substantial decline aligns with Rwanda’s multisectoral “First 1,000 Days” approach, led by the National Child Development Agency (NCDA), which targets nutrition, maternal care, WASH, agriculture, and social protection simultaneously (34, 35). These efforts have progressively addressed both the direct and structural drivers of chronic undernutrition, complementing the improvements noted in previous national surveys (23, 36, 37). Districts such as Kamonyi and Nyarugenge, which recorded the largest reductions, may benefit from intensified support through nutritional centers, strengthened CHW networks using tools such as the Child Length Mat, and targeted provision of fortified blended foods (FBFs) to vulnerable households. In contrast, persistent stunting in western districts such as Rubavu and Nyabihu is consistent with historical patterns of food insecurity and environmental enteric dysfunction in the highlands, underscoring the need for more targeted interventions (38).

The spatial trends in infectious diseases provide further insight into contextual factors influencing child health outcomes (33, 39). Persistent malaria hotspots in southern districts, particularly Gisagara, mirror earlier national malaria reports and regional evidence linking ecological suitability, vector adaptation, and climate variability to ongoing transmission despite national declines (23, 32, 39–42). Similarly, the clustering of respiratory infections in northern high-altitude districts reflects climatic conditions conducive to respiratory pathogen transmission and corroborates earlier analyses from Rwanda (43, 44). Meanwhile, the near-elimination of schistosomiasis aligns with long-running nationwide interventions, including preventive chemotherapy and snail-control campaigns (45). The multi-sectoral WASH initiatives, especially household water purification, quality monitoring, and expanded clean water access, likely also contribute to the exceptionally low schistosomiasis prevalence and the overall stability in diarrheal disease patterns observed across districts (41). Because these infectious disease indicators were derived from health facility consultations, observed geographical differences may reflect both underlying transmission and differences in healthcare access, care-seeking behavior, diagnostic practices, and reporting completeness across districts.

The association analyses indicate that stunting was not consistently associated with the selected disease indicators at the national district-level scale, whereas it’s shaped by complex, multifactorial interactions (6, 7). While no infectious disease showed a consistent relationship with stunting across all years, the emergence of a significant correlation between diarrheal diseases and stunting in 2024 aligns with findings from other studies on interlinkages between diarrheal and impaired nutrient absorption and linear growth faltering (46, 47). Rwanda’s WASH-focused components, including hygiene promotion, improved water quality, and the installation of clean water systems in schools and health facilities, directly address these risks and remain essential to sustaining declines in stunting. The relationship between infectious diseases and stunting should also be interpreted as potentially bidirectional. Recurrent infections may contribute to growth faltering by reducing appetite, impairing nutrient absorption, increasing metabolic demands, and promoting systemic or intestinal inflammation (32, 39, 46, 47). Conversely, children who are already stunted may be more susceptible to infections because chronic undernutrition can impair mucosal barrier function, innate and adaptive immune responses, and recovery from infectious episodes (32, 39, 48). This creates a possible vicious cycle in which undernutrition increases infection risk, while repeated infections may further worsen nutritional status and linear growth (39, 48). However, the ecological design of the present study does not allow determination of the direction of this relationship. Because stunting and infectious disease records were aggregated at the district level and were not linked to the same individual children, the observed associations should be interpreted as district-level ecological patterns rather than as evidence that infections caused stunting or that stunting increased infection risk in individual children.

The absence of nation-wide significant associations between stunting and infectious diseases among < 2 years in Rwanda contrasts findings from studies in other countries which suggest that disease incidence and stunting combined with structural determinants such as diet, maternal education, and poverty contribute positively to the incidence of stunting and chronic growth deficits (8–12, 32). However, at the district level, we observed an association of malaria with stunting in selected districts, namely Rutsiro, Musanze, Nyamagabe, Nyaruguru, Rubavu, and Kamonyi, all showing high positive correlation values of malaria association with stunting; Pearson’s r = < 0.80. Similarly, for diarrheal diseases, which showed a strong positive association with stunting in Gisagara district; Pearson’s r = < 0.90. As well as for respiratory diseases, the association with stunting was most nuanced in Nyamasheke, Nyarugenge, Kamonyi, and Muhanga; Pearson’s r = < 0.90. Therefore, it’s important to consider infectious diseases association with stunting in regional contexts such as ecological or environmental factors that further influence infectious diseases risk, including disease vectors emergence, distribution, and population exposure (49–51). Understanding these interlinked associations can further help design and implement region-specific targeted interventions to reduce the incidence and prevalence of stunting.

The strong negative district-level correlations observed in the stratified analyses warrant careful interpretation. These relationships likely reflect underlying contextual factors including household income, urbanization, access to care, and environmental conditions rather than direct causal effects. For instance, districts with high population density and urbanization (such as Gasabo and Kicukiro) demonstrated both lower stunting and lower infectious disease prevalence, consistent with the protective effects of improved living conditions, better water infrastructure, and higher maternal education (52). Therefore, these negative correlations are best interpreted as ecological signals of district-level contextual variation rather than causal or protective effects.

The multivariable model further emphasizes the importance of structural and socioeconomic conditions, particularly population density, which was inversely associated with stunting; *β* = −0.00517, *p* = 0.0004. Higher-density and urban districts benefit from improved access to diverse foods, maternal health services, WASH infrastructure, and economic opportunities. This aligns with the broader social protection and economic empowerment components of Rwanda’s anti-stunting program, including cash transfers through the Vision 2020 Umurenge Programme (VUP), public work initiatives that boost household income, and expanded Early Childhood Development (ECD) centers that integrate nutrition, stimulation, and growth monitoring. These findings correspond with previous analyses demonstrating that poverty alleviation, household diet diversification, and maternal empowerment play central roles in reducing child linear growth failure (32, 39).

Despite the absence of consistent nationwide associations, the district-level positive correlations observed in selected areas have important programmatic implications. Districts where malaria and stunting showed strong positive correlations, such as Rutsiro, Gisagara, Musanze, Nyamagabe, Rubavu, Bugesera, and Kamonyi, may benefit from integrated malaria–nutrition interventions targeting children under 2 years and pregnant women, particularly because previous studies have linked malaria infection with impaired growth, anemia, and increased risk of stunting or wasting in young children (8–12). These interventions could include strengthened malaria testing and treatment, insecticide-treated net use, seasonal or focal vector-control activities where appropriate, anemia screening, nutrition counseling, and growth monitoring in health facilities and community health workers (32). Similarly, the strong positive correlation between diarrheal diseases and stunting in Gisagara highlights the need to reinforce WASH interventions, household water treatment, oral rehydration and zinc management, nutrition counseling during and after diarrheal episodes, and follow-up of children with repeated diarrhea, given the established links between diarrheal illness, environmental enteric dysfunction, impaired nutrient absorption, and linear growth faltering (46, 47). For districts such as Nyamasheke, where respiratory infections were positively correlated with stunting, integrated management should include early identification and treatment of respiratory illness, assessment of nutritional status during sick-child visits, caregiver counseling, vaccination strengthening, and referral of children with recurrent illness or growth faltering, as undernutrition and stunting have been associated with poorer respiratory infection outcomes and delayed recovery in children (13–15). These district-specific patterns should not be interpreted causally, but they can support risk-based planning by identifying locations where nutrition services and infection-control activities should be jointly strengthened (32, 39, 51).

The agriculture and food security pillar of the national strategy, particularly the promotion of animal-source foods, biofortified crop distribution, and household kitchen gardens, likely contributed to nutritional improvements reflected in the declining stunting trends. These interventions enhance dietary diversity, address micronutrient deficiencies, and improve resilience in food-insecure districts (53). Moreover, Rwanda’s recent “Hehe n’Igwingira” social and behavior change campaign, together with the Inshuti z’Umuryango community volunteer network, has amplified community awareness of nutrition practices, early childcare, and infection prevention. Such behavioral interventions are essential for the uptake of multisectoral programs and may partly explain the strong progress observed in districts with high community engagement (54, 55).

### 4.1 Study limitations

This study is subject to several limitations due to reliance on the DHIS-2, which introduces challenges regarding data completeness and consistency; for instance, in this study, the DHIS-2 dataset lacks stunting surveillance data for 2021 due to COVID-19. Therefore, apparent trends between 2020 and 2022 should be interpreted with caution, as they may hide pandemic-related shifts in nutrition, health-seeking, infection reporting, or service access. This further affects the validity of year-on-year comparisons. Furthermore, the DHIS-2 lacks individualized health data, as some children might be stunted but not reporting infectious diseases or vice versa. Therefore, they reflect the proportion of reported diagnoses among children under 2 years who accessed health facilities in a given district and year, rather than the true population prevalence of infection. Districts with lower healthcare access, lower care-seeking behavior, or incomplete reporting may appear to have lower infectious disease burden even when community transmission is present. The DHIS-2 system often utilizes syndromic reporting or health worker-observed symptoms rather than definitive clinical diagnoses for the diseases under investigation. Methodologically, stunting is a cumulative, long-term outcome, and several of the factors examined may be influenced by reverse causality or residual confounding. Survival bias is also possible, as the analysis includes only children who lived to the time of the survey, potentially excluding the most vulnerable.

Additionally, the study uses variables that serve as imperfect proxies for complex underlying determinants; for example, population density does not fully account for the intricate socioeconomic and infrastructural factors that influence child growth. High degrees of co-linearity among closely related indicators such as water access, sanitation, and broader development metrics may further obscure specific relationships and lead to an underestimation of individual effects. As well, unmeasured confounding remains a significant concern, as data on critical factors such as paternal education, household food security, and dietary diversity were unavailable for inclusion. Consequently, these results should be interpreted as evidence of observational associations rather than definitive proof of causality.

## 5 Conclusion

Using aggregated data at district level and two distinct data sets, this study shows that Rwanda’s progress in reducing stunting is not associated with prevalence rates of the most important infectious diseases: diarrhea, malaria or respiratory infections among children: The infectious disease control remains vital for child wellbeing, Rwanda’s progress in reducing stunting is primarily driven by integrated, multisectoral policies that simultaneously address health, nutrition, food security, social protection, and WASH. The persistent geographic disparities in stunting and disease burden highlight areas requiring intensified, district-tailored investments, particularly in the western highlands and southern malaria-endemic regions. Continued strengthening of Rwanda’s multisectoral coordination, community engagement, and data-driven targeting will be critical for sustaining these gains and accelerating progress toward national and global nutrition targets.

## Data Availability

Publicly available datasets were analyzed in this study. This data can be found here: the datasets used and/or analyzed during the current study are available from the corresponding author on reasonable request (email: ntawuseleman@gmail.com).

## Footnotes

1
https://rbc.gov.rw/mat/
