# Supplementary material for: District-level ecological analysis of the association between stunting and infectious diseases among children under 2 years in Rwanda, 2020–2024
Source: Front Public Health. 2026 Aug 4;14:1855873. doi: 10.3389/fpubh.2026.1855873 (PMC13481546; doi:10.3389/fpubh.2026.1855873)
Supplement: Supplementary file 1 [file Supplementary_file_1.DOCX]

**Supplementary File 1:**

**Table S1:** Stunting counts and prevalence among under 2 years children in Rwanda

| **Year/District** | **Total Screened** | **Stunting cases** | **Prevalence (95% CI)** |
| --- | --- | --- | --- |
| **2020** | **377,361** | **140,036** | **33.1% [95% (33.0% - 33.3%)]** |
| Bugesera | 11,612 | 5,193 | 44.7% (95% CI: 43.8% - 45.6%) |
| Burera | 11,470 | 4,290 | 37.4% (95% CI: 38.3% - 36.5%) |
| Gakenke | 14,354 | 5,008 | 34.9% (95% CI: 35.7% - 34.1%) |
| Gasabo | 3,899 | 1,255 | 32.2% (95% CI: 33.7% - 30.7%) |
| Gatsibo | 20,277 | 7,252 | 35.8% (95% CI: 36.4% - 35.1%) |
| Gicumbi | 15,702 | 5,635 | 35.9% (95% CI: 36.6% - 35.1%) |
| Gisagara | 12,186 | 4,530 | 37.2% (95% CI: 38.0% - 36.3%) |
| Huye | 10,452 | 3,222 | 30.8% (95% CI: 31.7% - 29.9%) |
| Kamonyi | 9,737 | 3,416 | 35.1% (95% CI: 36.0% - 34.1%) |
| Karongi | 13,268 | 4,742 | 35.7% (95% CI: 36.6% - 34.9%) |
| Kayonza | 11,518 | 3,830 | 33.3% (95% CI: 34.1% - 32.4%) |
| Kicukiro | 8,751 | 2,283 | 26.1% (95% CI: 27.0% - 25.2%) |
| Kirehe | 13,079 | 4,454 | 34.1% (95% CI: 34.9% - 33.2%) |
| Muhanga | 9,083 | 3,697 | 40.7% (95% CI: 41.7% - 39.7%) |
| Musanze | 16,336 | 6,684 | 40.9% (95% CI: 41.7% - 40.2%) |
| Ngoma | 12,668 | 4,527 | 35.7% (95% CI: 36.6% - 34.9%) |
| Ngororero | 12,696 | 5,553 | 43.7% (95% CI: 44.6% - 42.9%) |
| Nyabihu | 9,615 | 3,130 | 32.6% (95% CI: 33.5% - 31.6%) |
| Nyagatare | 22,351 | 9,002 | 40.3% (95% CI: 40.9% - 39.6%) |
| Nyamagabe | 11,595 | 4,253 | 36.7% (95% CI: 37.6% - 35.8%) |
| Nyamasheke | 16,415 | 7,396 | 45.1% (95% CI: 45.8% - 44.3%) |
| Nyanza | 8,176 | 2,164 | 26.5% (95% CI: 27.4% - 25.5%) |
| Nyarugenge | 8,280 | 2,780 | 33.6% (95% CI: 34.6% - 32.6%) |
| Nyaruguru | 13,003 | 4,390 | 33.8% (95% CI: 34.6% - 32.9%) |
| Rubavu | 17,067 | 6,769 | 39.7% (95% CI: 40.4% - 38.9%) |
| Ruhango | 13,088 | 5,286 | 40.4% (95% CI: 41.2% - 39.5%) |
| Rulindo | 11,223 | 4,603 | 41.0% (95% CI: 41.9% - 40.1%) |
| Rusizi | 11,952 | 4,711 | 39.4% (95% CI: 40.3% - 38.5%) |
| Rutsiro | 13,791 | 6,156 | 44.6% (95% CI: 45.5% - 43.8%) |
| Rwamagana | 13,717 | 3,825 | 27.9% (95% CI: 28.6% - 27.1%) |
| **2022** | **297,856** | **99,957** | **33.6% (95% CI: 33.7% - 33.4%)** |
| Bugesera | 8,898 | 3,145 | 35.3% (95% CI: 36.3% - 34.4%) |
| Burera | 8,198 | 2,706 | 33.0% (95% CI: 34.0% - 32.0%) |
| Gakenke | 9,296 | 3,043 | 32.7% (95% CI: 33.7% - 31.8%) |
| Gasabo | 10,949 | 1,627 | 14.9% (95% CI: 15.5% - 14.2%) |
| Gatsibo | 9,026 | 2,493 | 27.6% (95% CI: 28.5% - 26.7%) |
| Gicumbi | 12,954 | 3,596 | 27.8% (95% CI: 28.5% - 27.0%) |
| Gisagara | 10,167 | 3,695 | 36.3% (95% CI: 37.3% - 35.4%) |
| Huye | 10,534 | 4,058 | 38.5% (95% CI: 39.5% - 37.6%) |
| Kamonyi | 7,664 | 1,589 | 20.7% (95% CI: 21.6% - 19.8%) |
| Karongi | 9,381 | 2,572 | 27.4% (95% CI: 28.3% - 26.5%) |
| Kayonza | 10,482 | 2,983 | 28.5% (95% CI: 29.3% - 27.6%) |
| Kicukiro | 4,713 | 815 | 17.3% (95% CI: 18.4% - 16.2%) |
| Kirehe | 10,956 | 3,566 | 32.5% (95% CI: 33.4% - 31.7%) |
| Muhanga | 5,058 | 1,579 | 31.2% (95% CI: 32.5% - 29.9%) |
| Musanze | 8,719 | 2,826 | 32.4% (95% CI: 33.4% - 31.4%) |
| Ngoma | 12,681 | 4,546 | 35.8% (95% CI: 36.7% - 35.0%) |
| Ngororero | 7,779 | 3,294 | 42.3% (95% CI: 43.4% - 41.2%) |
| Nyabihu | 8,290 | 2,781 | 33.5% (95% CI: 34.6% - 32.5%) |
| Nyagatare | 22,371 | 8,788 | 39.3% (95% CI: 39.9% - 38.6%) |
| Nyamagabe | 10,216 | 3,581 | 35.1% (95% CI: 36.0% - 34.1%) |
| Nyamasheke | 9,716 | 3,309 | 34.1% (95% CI: 35.0% - 33.1%) |
| Nyanza | 5,803 | 1,362 | 23.5% (95% CI: 24.6% - 22.4%) |
| Nyarugenge | 5,675 | 1,716 | 30.2% (95% CI: 31.4% - 29.0%) |
| Nyaruguru | 8,840 | 3,015 | 34.1% (95% CI: 35.1% - 33.1%) |
| Rubavu | 16,255 | 7,670 | 47.2% (95% CI: 48.0% - 46.4%) |
| Ruhango | 13,121 | 5,302 | 40.4% (95% CI: 41.2% - 39.6%) |
| Rulindo | 6,527 | 2,263 | 34.7% (95% CI: 35.8% - 33.5%) |
| Rusizi | 11,943 | 4,561 | 38.2% (95% CI: 39.1% - 37.3%) |
| Rutsiro | 7,924 | 3,731 | 47.1% (95% CI: 48.2% - 46.0%) |
| Rwamagana | 13,720 | 3,745 | 27.3% (95% CI: 28.0% - 26.6%) |
| **2023** | **368,729** | **92,213** | **25.0% (95% CI: 25.1% - 24.9%)** |
| Bugesera | 20,039 | 5,816 | 29.0% (95% CI: 29.7% - 28.4%) |
| Burera | 11,988 | 3,559 | 29.7% (95% CI: 30.5% - 28.9%) |
| Gakenke | 12,661 | 3,327 | 26.3% (95% CI: 27.0% - 25.5%) |
| Gasabo | 10,054 | 2,683 | 26.7% (95% CI: 27.6% - 25.8%) |
| Gatsibo | 14,091 | 3,312 | 23.5% (95% CI: 24.2% - 22.8%) |
| Gicumbi | 14,072 | 3,781 | 26.9% (95% CI: 27.6% - 26.1%) |
| Gisagara | 14,368 | 3,204 | 22.3% (95% CI: 23.0% - 21.6%) |
| Huye | 12,001 | 1,985 | 16.5% (95% CI: 17.2% - 15.9%) |
| Kamonyi | 11,988 | 2,531 | 21.1% (95% CI: 21.8% - 20.4%) |
| Karongi | 10,949 | 2,755 | 25.2% (95% CI: 26.0% - 24.3%) |
| Kayonza | 10,248 | 2,796 | 27.3% (95% CI: 28.1% - 26.4%) |
| Kicukiro | 8,125 | 1,672 | 20.6% (95% CI: 21.5% - 19.7%) |
| Kirehe | 15,617 | 2,199 | 14.1% (95% CI: 14.6% - 13.5%) |
| Muhanga | 9,824 | 1,850 | 18.8% (95% CI: 19.6% - 18.1%) |
| Musanze | 12,995 | 3,576 | 27.5% (95% CI: 28.3% - 26.8%) |
| Ngoma | 14,341 | 3,958 | 27.6% (95% CI: 28.3% - 26.9%) |
| Ngororero | 10,652 | 2,482 | 23.3% (95% CI: 24.1% - 22.5%) |
| Nyabihu | 9,181 | 3,101 | 33.8% (95% CI: 34.7% - 32.8%) |
| Nyagatare | 18,237 | 5,455 | 29.9% (95% CI: 30.6% - 29.2%) |
| Nyamagabe | 11,503 | 3,026 | 26.3% (95% CI: 27.1% - 25.5%) |
| Nyamasheke | 11,571 | 3,839 | 33.2% (95% CI: 34.0% - 32.3%) |
| Nyanza | 11,393 | 2,918 | 25.6% (95% CI: 26.4% - 24.8%) |
| Nyarugenge | 7,278 | 848 | 11.7% (95% CI: 12.4% - 10.9%) |
| Nyaruguru | 11,289 | 2,407 | 21.3% (95% CI: 22.1% - 20.6%) |
| Rubavu | 15,167 | 3,076 | 20.3% (95% CI: 20.9% - 19.6%) |
| Ruhango | 10,845 | 2,707 | 25.0% (95% CI: 25.8% - 24.1%) |
| Rulindo | 8,746 | 2,614 | 29.9% (95% CI: 30.8% - 28.9%) |
| Rusizi | 13,749 | 4,072 | 29.6% (95% CI: 30.4% - 28.9%) |
| Rutsiro | 10,603 | 3,705 | 34.9% (95% CI: 35.9% - 34.0%) |
| Rwamagana | 15,154 | 2,959 | 19.5% (95% CI: 20.2% - 18.9%) |
| **2024** | **384,702** | **76,872** | **21.7% (95% CI: 22.1% - 20.9%)** |
| Bugesera | 17,763 | 4,010 | 22.6% (95% CI: 23.2% - 22.0%) |
| Burera | 10,915 | 3,163 | 29.0% (95% CI: 29.8% - 28.1%) |
| Gakenke | 12,309 | 2,958 | 24.0% (95% CI: 24.8% - 23.3%) |
| Gasabo | 14,566 | 2,721 | 18.7% (95% CI: 19.3% - 18.0%) |
| Gatsibo | 12,575 | 3,134 | 24.9% (95% CI: 25.7% - 24.2%) |
| Gicumbi | 14,848 | 2,834 | 19.1% (95% CI: 19.7% - 18.5%) |
| Gisagara | 13,863 | 2,460 | 17.7% (95% CI: 18.4% - 17.1%) |
| Huye | 12,578 | 2,008 | 16.0% (95% CI: 16.6% - 15.3%) |
| Kamonyi | 13,156 | 1,240 | 9.4% (95% CI: 9.9% - 8.9%) |
| Karongi | 10,598 | 2,678 | 25.3% (95% CI: 26.1% - 24.4%) |
| Kayonza | 11,349 | 2,455 | 21.6% (95% CI: 22.4% - 20.9%) |
| Kicukiro | 9,744 | 1,262 | 13.0% (95% CI: 13.6% - 12.3%) |
| Kirehe | 15,973 | 1,905 | 11.9% (95% CI: 12.4% - 11.4%) |
| Muhanga | 10,710 | 1,305 | 12.2% (95% CI: 12.8% - 11.6%) |
| Musanze | 14,298 | 2,988 | 20.9% (95% CI: 21.6% - 20.2%) |
| Ngoma | 15,945 | 1,799 | 11.3% (95% CI: 11.8% - 10.8%) |
| Ngororero | 11,326 | 2,771 | 24.5% (95% CI: 25.3% - 23.7%) |
| Nyabihu | 11,352 | 3,560 | 31.4% (95% CI: 32.2% - 30.5%) |
| Nyagatare | 17,858 | 3,811 | 21.3% (95% CI: 21.9% - 20.7%) |
| Nyamagabe | 12,935 | 2,449 | 18.9% (95% CI: 19.6% - 18.3%) |
| Nyamasheke | 11,611 | 2,715 | 23.4% (95% CI: 24.2% - 22.6%) |
| Nyanza | 6,307 | 631 | 10.0% (95% CI: 10.7% - 9.3%) |
| Nyarugenge | 9,729 | 766 | 7.9% (95% CI: 8.4% - 7.3%) |
| Nyaruguru | 12,131 | 3,444 | 28.4% (95% CI: 29.2% - 27.6%) |
| Rubavu | 15,014 | 4,731 | 31.5% (95% CI: 32.3% - 30.8%) |
| Ruhango | 10,784 | 2,291 | 21.2% (95% CI: 22.0% - 20.5%) |
| Rulindo | 10,690 | 2,559 | 23.9% (95% CI: 24.7% - 23.1%) |
| Rusizi | 15,412 | 3,033 | 19.7% (95% CI: 20.3% - 19.1%) |
| Rutsiro | 11,683 | 3,265 | 27.9% (95% CI: 28.8% - 27.1%) |
| Rwamagana | 16,680 | 1,926 | 11.5% (95% CI: 12.0% - 11.1%) |

**Table S2:** Percentage change in stunting prevalence between 2020-2024 among under 2 years children in Rwanda

| **District** | **2020 Stunting Prevalence** | **2024 Stunting Prevalence** | **% change in Stunting Prevalence** |
| --- | --- | --- | --- |
| Bugesera | 44.7% (95% CI: 43.80% - 45.60%) | 22.6% (95% CI: 22.0% - 23.20%) | -49.5% (95% CI: -49.9% - -49.20%) |
| Burera | 37.4% (95% CI: 36.50% - 38.30%) | 29.0% (95% CI: 28.1% - 29.80%) | -22.5% (95% CI: -23.0% - -22.10%) |
| Gakenke | 34.9% (95% CI: 34.10% - 35.70%) | 24.0% (95% CI: 23.3% - 24.80%) | -31.1% (95% CI: -31.8% - -30.50%) |
| Gasabo | 32.2% (95% CI: 30.70% - 33.70%) | 18.7% (95% CI: 18.0% - 19.30%) | -42.0% (95% CI: -41.3% - -42.60%) |
| Gatsibo | 35.8% (95% CI: 35.10% - 36.40%) | 24.9% (95% CI: 24.2% - 25.70%) | -30.3% (95% CI: -31.2% - -29.50%) |
| Gicumbi | 35.9% (95% CI: 35.10% - 36.60%) | 19.1% (95% CI: 18.5% - 19.70%) | -46.8% (95% CI: -47.5% - -46.20%) |
| Gisagara | 37.2% (95% CI: 36.30% - 38.00%) | 17.7% (95% CI: 17.1% - 18.40%) | -52.3% (95% CI: -52.9% - -51.70%) |
| Huye | 30.8% (95% CI: 29.90% - 31.70%) | 16.0% (95% CI: 15.3% - 16.60%) | -48.2% (95% CI: -48.8% - -47.60%) |
| Kamonyi | 35.1% (95% CI: 34.10% - 36.00%) | 9.4% (95% CI: 8.9% - 9.90%) | -73.1% (95% CI: -73.9% - -72.50%) |
| Karongi | 35.7% (95% CI: 34.90% - 36.60%) | 25.3% (95% CI: 24.4% - 26.10%) | -29.3% (95% CI: -30.0% - -28.60%) |
| Kayonza | 33.3% (95% CI: 32.40% - 34.10%) | 21.6% (95% CI: 20.9% - 22.40%) | -34.9% (95% CI: -35.6% - -34.40%) |
| Kicukiro | 26.1% (95% CI: 25.20% - 27.00%) | 13.0% (95% CI: 12.3% - 13.60%) | -50.4% (95% CI: -51.2% - -49.60%) |
| Kirehe | 34.1% (95% CI: 33.20% - 34.90%) | 11.9% (95% CI: 11.4% - 12.40%) | -65.0% (95% CI: -65.6% - -64.40%) |
| Muhanga | 40.7% (95% CI: 39.70% - 41.70%) | 12.2% (95% CI: 11.6% - 12.80%) | -70.1% (95% CI: -70.9% - -69.30%) |
| Musanze | 40.9% (95% CI: 40.20% - 41.70%) | 20.9% (95% CI: 20.2% - 21.60%) | -48.9% (95% CI: -49.6% - -48.20%) |
| Ngoma | 35.7% (95% CI: 34.90% - 36.60%) | 11.3% (95% CI: 10.8% - 11.80%) | -68.4% (95% CI: -69.1% - -67.80%) |
| Ngororero | 43.7% (95% CI: 42.90% - 44.60%) | 24.5% (95% CI: 23.7% - 25.30%) | -44.1% (95% CI: -44.8% - -43.40%) |
| Nyabihu | 32.6% (95% CI: 31.60% - 33.50%) | 31.4% (95% CI: 30.5% - 32.20%) | -3.7% (95% CI: -3.5% - -3.80%) |
| Nyagatare | 40.3% (95% CI: 39.60% - 40.90%) | 21.3% (95% CI: 20.7% - 21.90%) | -47.0% (95% CI: -47.7% - -46.40%) |
| Nyamagabe | 36.7% (95% CI: 35.80% - 37.60%) | 18.9% (95% CI: 18.3% - 19.60%) | -48.4% (95% CI: -49.0% - -47.80%) |
| Nyamasheke | 45.1% (95% CI: 44.30% - 45.80%) | 23.4% (95% CI: 22.6% - 24.20%) | -48.1% (95% CI: -48.9% - -47.30%) |
| Nyanza | 26.5% (95% CI: 25.50% - 27.40%) | 10.0% (95% CI: 9.3% - 10.70%) | -62.2% (95% CI: -63.7% - -60.80%) |
| Nyarugenge | 33.6% (95% CI: 32.60% - 34.60%) | 7.9% (95% CI: 7.3% - 8.40%) | -76.5% (95% CI: -77.5% - -75.70%) |
| Nyaruguru | 33.8% (95% CI: 32.90% - 34.60%) | 28.4% (95% CI: 27.6% - 29.20%) | -15.9% (95% CI: -16.3% - -15.60%) |
| Rubavu | 39.7% (95% CI: 38.90% - 40.40%) | 31.5% (95% CI: 30.8% - 32.30%) | -20.6% (95% CI: -21.0% - -20.20%) |
| Ruhango | 40.4% (95% CI: 39.50% - 41.20%) | 21.2% (95% CI: 20.5% - 22.00%) | -47.4% (95% CI: -48.2% - -46.60%) |
| Rulindo | 41.0% (95% CI: 40.10% - 41.90%) | 23.9% (95% CI: 23.1% - 24.70%) | -41.6% (95% CI: -42.3% - -41.00%) |
| Rusizi | 39.4% (95% CI: 38.50% - 40.30%) | 19.7% (95% CI: 19.1% - 20.30%) | -50.1% (95% CI: -50.6% - -49.60%) |
| Rutsiro | 44.6% (95% CI: 43.80% - 45.50%) | 27.9% (95% CI: 27.1% - 28.80%) | -37.4% (95% CI: -38.1% - -36.70%) |
| Rwamagana | 27.9% (95% CI: 27.10% - 28.60%) | 11.5% (95% CI: 11.1% - 12.00%) | -58.6% (95% CI: -59.2% - -58.00%) |
| **Rwanda** | **33.1% (95% CI: 33.0% - 33.3%)** | **21.7% (95% CI: 20.9% - 22.1%)** | **-34.4% (95% CI: -36.7% - -33.6%])** |

**Table S3:** The number of infectious diseases cases and prevalence among under 2 years children in Rwanda

| **District** | **Under 2 years population** | **Malaria cases** | **Schistosoma cases** | **Diarrhea cases** | **Respiratory cases** | **Malaria Prevalence %** | **Schistosoma Prevalence %** | **Diarrhea Prevalence %** | **Respiratory Prevalence %** |
| --- | --- | --- | --- | --- | --- | --- | --- | --- | --- |
| **2020** |  |  |  |  |  |  |  |  |  |
| Bugesera | 11,612 | 2,066 | 0 | 574 | 561 | 17.8 | 0.0 | 4.9 | 4.8 |
| Burera | 11,470 | 422 | 0 | 822 | 1,448 | 3.7 | 0.0 | 7.2 | 12.6 |
| Gakenke | 14,354 | 368 | 0 | 840 | 1,007 | 2.6 | 0.0 | 5.9 | 7.0 |
| Gasabo | 3,899 | 191 | 0 | 106 | 105 | 4.9 | 0.0 | 2.7 | 2.7 |
| Gatsibo | 20,277 | 276 | 0 | 954 | 1,158 | 1.4 | 0.0 | 4.7 | 5.7 |
| Gicumbi | 15,702 | 1,320 | 0 | 910 | 1,485 | 8.4 | 0.0 | 5.8 | 9.5 |
| Gisagara | 12,186 | 4,037 | 0 | 916 | 1,252 | 33.1 | 0.0 | 7.5 | 10.3 |
| Huye | 10,452 | 918 | 0 | 569 | 1,020 | 8.8 | 0.0 | 5.4 | 9.8 |
| Kamonyi | 9,737 | 1,080 | 0 | 234 | 393 | 11.1 | 0.0 | 2.4 | 4.0 |
| Karongi | 13,268 | 291 | 0 | 502 | 712 | 2.2 | 0.0 | 3.8 | 5.4 |
| Kayonza | 11,518 | 765 | 0 | 292 | 234 | 6.6 | 0.0 | 2.5 | 2.0 |
| Kicukiro | 8,751 | 572 | 0 | 187 | 301 | 6.5 | 0.0 | 2.1 | 3.4 |
| Kirehe | 13,079 | 507 | 0 | 782 | 1,215 | 3.9 | 0.0 | 6.0 | 9.3 |
| Muhanga | 9,083 | 231 | 0 | 322 | 638 | 2.5 | 0.0 | 3.5 | 7.0 |
| Musanze | 16,336 | 381 | 0 | 866 | 1,184 | 2.3 | 0.0 | 5.3 | 7.3 |
| Ngoma | 12,668 | 89 | 0 | 454 | 562 | 0.7 | 0.0 | 3.6 | 4.4 |
| Ngororero | 12,696 | 359 | 0 | 338 | 272 | 2.8 | 0.0 | 2.7 | 2.1 |
| Nyabihu | 9,615 | 17 | 0 | 515 | 765 | 0.2 | 0.0 | 5.4 | 8.0 |
| Nyagatare | 22,351 | 1,264 | 0 | 781 | 946 | 5.7 | 0.0 | 3.5 | 4.2 |
| Nyamagabe | 11,595 | 1,951 | 0 | 483 | 550 | 16.8 | 0.0 | 4.2 | 4.7 |
| Nyamasheke | 16,415 | 978 | 0 | 543 | 1,299 | 6.0 | 0.0 | 3.3 | 7.9 |
| Nyanza | 8,176 | 984 | 0 | 336 | 588 | 12.0 | 0.0 | 4.1 | 7.2 |
| Nyarugenge | 8,280 | 380 | 0 | 288 | 274 | 4.6 | 0.0 | 3.5 | 3.3 |
| Nyaruguru | 13,003 | 1,384 | 0 | 863 | 784 | 10.6 | 0.0 | 6.6 | 6.0 |
| Rubavu | 17,067 | 220 | 0 | 666 | 644 | 1.3 | 0.0 | 3.9 | 3.8 |
| Ruhango | 13,088 | 1,553 | 0 | 478 | 778 | 11.9 | 0.0 | 3.7 | 5.9 |
| Rulindo | 11,223 | 259 | 0 | 496 | 586 | 2.3 | 0.0 | 4.4 | 5.2 |
| Rusizi | 11,952 | 389 | 0 | 305 | 465 | 3.3 | 0.0 | 2.6 | 3.9 |
| Rutsiro | 13,791 | 923 | 0 | 637 | 728 | 6.7 | 0.0 | 4.6 | 5.3 |
| Rwamagana | 13,717 | 747 | 1 | 474 | 978 | 5.4 | 0.0 | 3.5 | 7.1 |
| **2022** |  |  |  |  |  |  |  |  |  |
| Bugesera | 8,898 | 376 | 0 | 448 | 593 | 4.2 | 0.0 | 5.0 | 6.7 |
| Burera | 8,198 | 515 | 0 | 653 | 1,331 | 6.3 | 0.0 | 8.0 | 16.2 |
| Gakenke | 9,296 | 241 | 0 | 733 | 679 | 2.6 | 0.0 | 7.9 | 7.3 |
| Gasabo | 10,949 | 1,339 | 0 | 279 | 394 | 12.2 | 0.0 | 2.5 | 3.6 |
| Gatsibo | 9,026 | 208 | 0 | 519 | 676 | 2.3 | 0.0 | 5.8 | 7.5 |
| Gicumbi | 12,954 | 1,250 | 0 | 1,020 | 2,035 | 9.6 | 0.0 | 7.9 | 15.7 |
| Gisagara | 10,167 | 994 | 0 | 770 | 1,146 | 9.8 | 0.0 | 7.6 | 11.3 |
| Huye | 10,534 | 422 | 0 | 686 | 1,011 | 4.0 | 0.0 | 6.5 | 9.6 |
| Kamonyi | 7,664 | 106 | 0 | 236 | 371 | 1.4 | 0.0 | 3.1 | 4.8 |
| Karongi | 9,381 | 252 | 0 | 453 | 425 | 2.7 | 0.0 | 4.8 | 4.5 |
| Kayonza | 10,482 | 80 | 0 | 340 | 311 | 0.8 | 0.0 | 3.2 | 3.0 |
| Kicukiro | 4,713 | 550 | 0 | 137 | 350 | 11.7 | 0.0 | 2.9 | 7.4 |
| Kirehe | 10,956 | 232 | 0 | 845 | 1,298 | 2.1 | 0.0 | 7.7 | 11.8 |
| Muhanga | 5,058 | 111 | 0 | 234 | 389 | 2.2 | 0.0 | 4.6 | 7.7 |
| Musanze | 8,719 | 182 | 0 | 488 | 1,069 | 2.1 | 0.0 | 5.6 | 12.3 |
| Ngoma | 12,681 | 26 | 0 | 586 | 714 | 0.2 | 0.0 | 4.6 | 5.6 |
| Ngororero | 7,779 | 319 | 0 | 273 | 604 | 4.1 | 0.0 | 3.5 | 7.8 |
| Nyabihu | 8,290 | 26 | 0 | 473 | 566 | 0.3 | 0.0 | 5.7 | 6.8 |
| Nyagatare | 22,371 | 532 | 0 | 1,164 | 1,794 | 2.4 | 0.0 | 5.2 | 8.0 |
| Nyamagabe | 10,216 | 1,265 | 0 | 597 | 560 | 12.4 | 0.0 | 5.8 | 5.5 |
| Nyamasheke | 9,716 | 504 | 0 | 386 | 527 | 5.2 | 0.0 | 4.0 | 5.4 |
| Nyanza | 5,803 | 214 | 0 | 283 | 539 | 3.7 | 0.0 | 4.9 | 9.3 |
| Nyarugenge | 5,675 | 550 | 0 | 194 | 187 | 9.7 | 0.0 | 3.4 | 3.3 |
| Nyaruguru | 8,840 | 1,080 | 0 | 722 | 942 | 12.2 | 0.0 | 8.2 | 10.7 |
| Rubavu | 16,255 | 197 | 0 | 629 | 1,358 | 1.2 | 0.0 | 3.9 | 8.4 |
| Ruhango | 13,121 | 208 | 0 | 722 | 967 | 1.6 | 0.0 | 5.5 | 7.4 |
| Rulindo | 6,527 | 431 | 0 | 317 | 553 | 6.6 | 0.0 | 4.8 | 8.5 |
| Rusizi | 11,943 | 327 | 0 | 424 | 471 | 2.7 | 0.0 | 3.5 | 3.9 |
| Rutsiro | 7,924 | 580 | 0 | 421 | 837 | 7.3 | 0.0 | 5.3 | 10.6 |
| Rwamagana | 13,720 | 254 | 0 | 520 | 1,043 | 1.9 | 0.0 | 3.8 | 7.6 |
| **2023** |  |  |  |  |  |  |  |  |  |
| Bugesera | 20,039 | 531 | 0 | 973 | 1,347 | 2.6 | 0.0 | 4.9 | 6.7 |
| Burera | 11,988 | 727 | 0 | 939 | 1,495 | 6.1 | 0.0 | 7.8 | 12.5 |
| Gakenke | 12,661 | 322 | 0 | 896 | 916 | 2.5 | 0.0 | 7.1 | 7.2 |
| Gasabo | 10,054 | 643 | 0 | 276 | 382 | 6.4 | 0.0 | 2.7 | 3.8 |
| Gatsibo | 14,091 | 104 | 0 | 690 | 869 | 0.7 | 0.0 | 4.9 | 6.2 |
| Gicumbi | 14,072 | 525 | 0 | 904 | 1,944 | 3.7 | 0.0 | 6.4 | 13.8 |
| Gisagara | 14,368 | 1,731 | 0 | 960 | 1,335 | 12.0 | 0.0 | 6.7 | 9.3 |
| Huye | 12,001 | 141 | 0 | 671 | 1,003 | 1.2 | 0.0 | 5.6 | 8.4 |
| Kamonyi | 11,988 | 63 | 0 | 373 | 672 | 0.5 | 0.0 | 3.1 | 5.6 |
| Karongi | 10,949 | 234 | 0 | 575 | 331 | 2.1 | 0.0 | 5.3 | 3.0 |
| Kayonza | 10,248 | 55 | 0 | 363 | 336 | 0.5 | 0.0 | 3.5 | 3.3 |
| Kicukiro | 8,125 | 603 | 0 | 279 | 657 | 7.4 | 0.0 | 3.4 | 8.1 |
| Kirehe | 15,617 | 443 | 0 | 1,174 | 1,708 | 2.8 | 0.0 | 7.5 | 10.9 |
| Muhanga | 9,824 | 270 | 0 | 433 | 805 | 2.7 | 0.0 | 4.4 | 8.2 |
| Musanze | 12,995 | 264 | 0 | 792 | 1,853 | 2.0 | 0.0 | 6.1 | 14.3 |
| Ngoma | 14,341 | 36 | 0 | 612 | 772 | 0.2 | 0.0 | 4.3 | 5.4 |
| Ngororero | 10,652 | 256 | 0 | 357 | 720 | 2.4 | 0.0 | 3.4 | 6.8 |
| Nyabihu | 9,181 | 28 | 0 | 581 | 838 | 0.3 | 0.0 | 6.3 | 9.1 |
| Nyagatare | 18,237 | 552 | 0 | 946 | 1,443 | 3.0 | 0.0 | 5.2 | 7.9 |
| Nyamagabe | 11,503 | 772 | 0 | 647 | 614 | 6.7 | 0.0 | 5.6 | 5.3 |
| Nyamasheke | 11,571 | 606 | 0 | 404 | 545 | 5.2 | 0.0 | 3.5 | 4.7 |
| Nyanza | 11,393 | 193 | 0 | 591 | 1,184 | 1.7 | 0.0 | 5.2 | 10.4 |
| Nyarugenge | 7,278 | 346 | 0 | 259 | 308 | 4.8 | 0.0 | 3.6 | 4.2 |
| Nyaruguru | 11,289 | 294 | 0 | 871 | 931 | 2.6 | 0.0 | 7.7 | 8.2 |
| Rubavu | 15,167 | 94 | 0 | 667 | 1,556 | 0.6 | 0.0 | 4.4 | 10.3 |
| Ruhango | 10,845 | 52 | 0 | 486 | 900 | 0.5 | 0.0 | 4.5 | 8.3 |
| Rulindo | 8,746 | 686 | 0 | 437 | 683 | 7.8 | 0.0 | 5.0 | 7.8 |
| Rusizi | 13,749 | 232 | 0 | 527 | 480 | 1.7 | 0.0 | 3.8 | 3.5 |
| Rutsiro | 10,603 | 398 | 0 | 633 | 1,289 | 3.8 | 0.0 | 6.0 | 12.2 |
| Rwamagana | 15,154 | 143 | 0 | 604 | 1,050 | 0.9 | 0.0 | 4.0 | 6.9 |
| **2024** |  |  |  |  |  |  |  |  |  |
| Bugesera | 17,763 | 853 | 0 | 781 | 977 | 4.8 | 0.0 | 4.4 | 5.5 |
| Burera | 10,915 | 300 | 0 | 815 | 1,159 | 2.8 | 0.0 | 7.5 | 10.6 |
| Gakenke | 12,309 | 141 | 0 | 844 | 826 | 1.1 | 0.0 | 6.9 | 6.7 |
| Gasabo | 14,566 | 1,151 | 0 | 284 | 323 | 7.9 | 0.0 | 2.0 | 2.2 |
| Gatsibo | 12,575 | 108 | 0 | 588 | 811 | 0.9 | 0.0 | 4.7 | 6.4 |
| Gicumbi | 14,848 | 418 | 0 | 859 | 1,784 | 2.8 | 0.0 | 5.8 | 12.0 |
| Gisagara | 13,863 | 3,940 | 0 | 867 | 761 | 28.4 | 0.0 | 6.3 | 5.5 |
| Huye | 12,578 | 148 | 0 | 615 | 813 | 1.2 | 0.0 | 4.9 | 6.5 |
| Kamonyi | 13,156 | 63 | 0 | 451 | 873 | 0.5 | 0.0 | 3.4 | 6.6 |
| Karongi | 10,598 | 253 | 0 | 594 | 437 | 2.4 | 0.0 | 5.6 | 4.1 |
| Kayonza | 11,349 | 179 | 0 | 347 | 139 | 1.6 | 0.0 | 3.1 | 1.2 |
| Kicukiro | 9,744 | 1,419 | 0 | 236 | 611 | 14.6 | 0.0 | 2.4 | 6.3 |
| Kirehe | 15,973 | 546 | 0 | 930 | 1,133 | 3.4 | 0.0 | 5.8 | 7.1 |
| Muhanga | 10,710 | 154 | 0 | 395 | 867 | 1.4 | 0.0 | 3.7 | 8.1 |
| Musanze | 14,298 | 223 | 0 | 890 | 1,829 | 1.6 | 0.0 | 6.2 | 12.8 |
| Ngoma | 15,945 | 61 | 0 | 647 | 579 | 0.4 | 0.0 | 4.1 | 3.6 |
| Ngororero | 11,326 | 115 | 0 | 349 | 689 | 1.0 | 0.0 | 3.1 | 6.1 |
| Nyabihu | 11,352 | 45 | 0 | 658 | 735 | 0.4 | 0.0 | 5.8 | 6.5 |
| Nyagatare | 17,858 | 968 | 0 | 819 | 1,255 | 5.4 | 0.0 | 4.6 | 7.0 |
| Nyamagabe | 12,935 | 816 | 0 | 743 | 667 | 6.3 | 0.0 | 5.7 | 5.2 |
| Nyamasheke | 11,611 | 1,692 | 0 | 424 | 530 | 14.6 | 0.0 | 3.7 | 4.6 |
| Nyanza | 6,307 | 226 | 0 | 360 | 534 | 3.6 | 0.0 | 5.7 | 8.5 |
| Nyarugenge | 9,729 | 681 | 0 | 358 | 475 | 7.0 | 0.0 | 3.7 | 4.9 |
| Nyaruguru | 12,131 | 399 | 0 | 702 | 750 | 3.3 | 0.0 | 5.8 | 6.2 |
| Rubavu | 15,014 | 94 | 0 | 1,008 | 1,169 | 0.6 | 0.0 | 6.7 | 7.8 |
| Ruhango | 10,784 | 72 | 0 | 530 | 910 | 0.7 | 0.0 | 4.9 | 8.4 |
| Rulindo | 10,690 | 315 | 0 | 503 | 852 | 2.9 | 0.0 | 4.7 | 8.0 |
| Rusizi | 15,412 | 476 | 0 | 760 | 573 | 3.1 | 0.0 | 4.9 | 3.7 |
| Rutsiro | 11,683 | 198 | 0 | 640 | 1,022 | 1.7 | 0.0 | 5.5 | 8.8 |
| Rwamagana | 16,680 | 194 | 0 | 552 | 560 | 1.2 | 0.0 | 3.3 | 3.4 |
